# Laparoscopic Management of Huge Ovarian Cysts

**DOI:** 10.1155/2013/380854

**Published:** 2013-05-08

**Authors:** A. Alobaid, A. Memon, S. Alobaid, L. Aldakhil

**Affiliations:** ^1^King Fahad Medical City, Women's Specialized Hospital, P.O. Box 59046, Riyadh 11525, Saudi Arabia; ^2^King Khaled University Hospital, King Saud University, P.O. Box 2925, Riyadh 11461, Saudi Arabia

## Abstract

*Objectives.* Huge ovarian cysts are conventionally managed by laparotomy. We present 5 cases with huge ovarian cysts managed by laparoscopic endoscopic surgery without any complications. *Materials and Methods.* We describe five patients who had their surgeries conducted in a tertiary care center in Riyadh, Saudi Arabia (King Fahad Medical City). *Results.* Patients age ranged between 19 and 69 years. Tumor markers were normal for all patients. The maximum diameter of all cysts ranged between 18 and 42 cm as measured by ultrasound. The cysts were unilocular; in some patients, there were fine septations. All patients had open-entry laparoscopy. After evaluation of the cyst capsule, the cysts were drained under laparoscopic guidance, 1–12 liters were drained from the cysts (mean 5.2 L), and then laparoscopic oophorectomy was done. The final histopathology reports confirmed benign serous cystadenoma in four patients and one patient had a benign mucinous cystadenoma. There was minimal blood loss during surgeries and with no complications for all patients. *Conclusion.* There is still no consensus for the size limitation of ovarian cysts decided to be a contraindication for laparoscopic management. With advancing techniques, proper patients selection, and availability of experts in gynecologic endoscopy, it is possible to remove giant cyst by laparoscopy.

## 1. Introduction

Ovarian neoplasms are a common clinical problem affecting women of all age groups. They are the fourth most common reason for gynecologic admission in the United States, and it has been estimated that approximately 10% of women in the United States will undergo surgical procedure for a suspected ovarian neoplasm during their lifetime [1].

Laparoscopy is considered the gold standard approach to manage benign ovarian cysts. The benefits of laparoscopy include reduced postoperative analgesic requirement, earlier mobilization reducing chances of deep venous thrombosis (DVT), cosmetic advantages, earlier discharge from the hospital, and return to normal activity.

A major factor that will make the gynecologic surgeon decide to perform a laparotomy is the size of the ovarian mass.

The definition of huge ovarian cysts is not well described in the literature. Some authors define large ovarian cysts as those that are more than 10 cm in diameter as measured by preoperative scans [2]. Others define large ovarian cysts as those that are reaching above the umbilicus [3].

Laparoscopic management of huge ovarian cysts has been described in previous case reports [4–11]. Despite this, most patients with huge ovarian cysts are managed by laparotomy.

The aim of our study is to evaluate the safety, effectiveness, and feasibility of operative laparoscopy in the management of huge ovarian cysts reaching above the umbilicus.

We present five patients with extremely large ovarian cysts that were all managed laparoscopically. 

## 2. Material and Methods

We describe five cases of huge ovarian cysts managed successfully by laparoscopy. All surgeries were performed in the Women's Specialized Hospital, King Fahad Medical City Hospital in Riyadh, by the principal author. The surgeries were performed between April 2009 and December 2010. 

If the history, physical examination, and radiological findings were in favour of a benign nature of the cysts, then the patient would be selected for endoscopic approach. Tumor markers were requested for all patients. Informed consent was taken for possible conversion to laparotomy in case of technical difficulties or if there is an incidental finding of malignancy. All surgeries were performed under general anaesthesia.

Open-entry laparoscopic technique (Hasson method) was used to avoid puncturing of cyst prior to the evaluation of the cyst that is done intraoperatively. The cyst wall was then inspected prior to its drainage. If there were no signs of malignancy, the cyst was then drained under laparoscopic guidance using a suction irrigation device. After that we proceeded with laparoscopic oophorectomy or cystectomy in the usual manner. 

**Figure 1:**
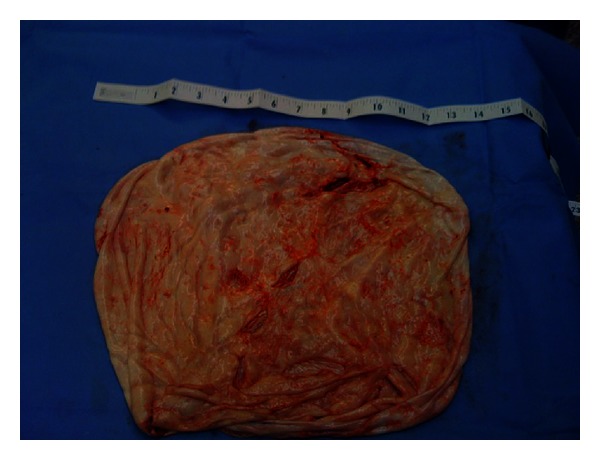
Huge ovarian serous cystadenoma specimen from patient 4.

The cyst was then removed using the suprapubic trocar after extending the incision to 1.5 cm (Figure 1). 

## 3. Results

**Figure 2:**
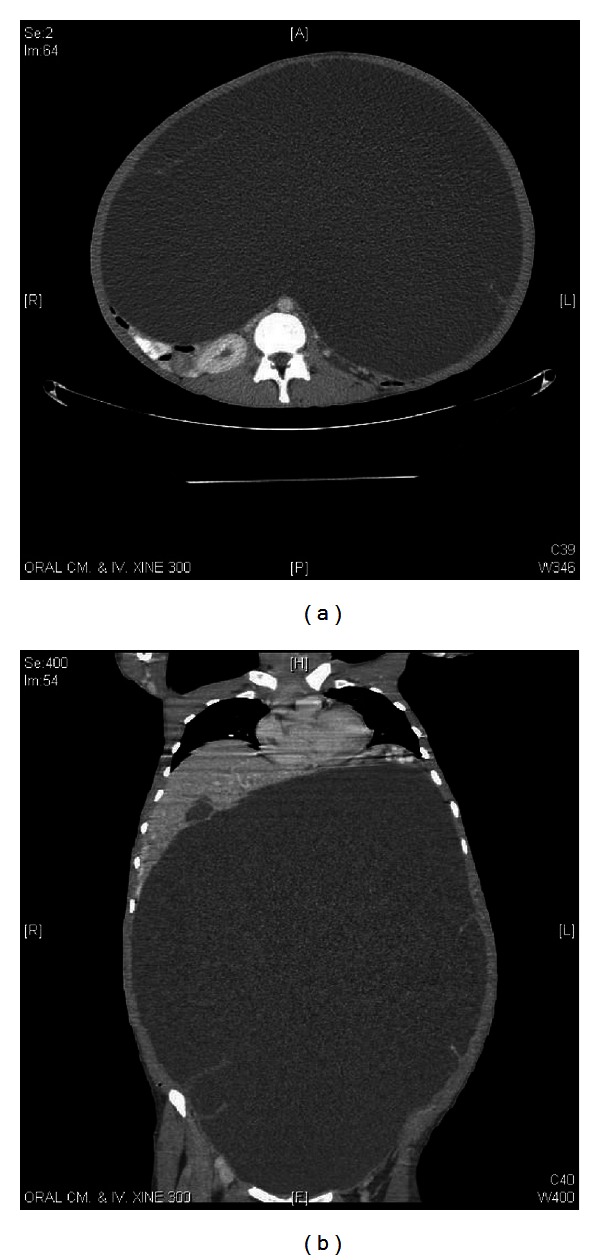
Axial and coronal computerized tomography (CT) scan images showing a huge ovarian cyst of patient 4.

All the patients had similar presentations that were nonspecific such as abdominal distention and discomfort. The mean age was 30.6 years (range 19–69 years) (Table 1). The patients had good general health. The third patient has had a third degree burn when she was a child and had abdominal skin graft. Otherwise, there were no previous surgeries done for all patients. The family history was negative for ovarian cancer in all patients. The examination revealed huge ovarian masses that were all reaching above the umbilicus, and in some patients it was reaching the xiphoid process (Figures 2(a) and 2(b)). For patients in their second and third decades of life, the tumor markers that were verified included CA-125, lactate dehydrogenase (LDH), alpha-feto-protein, and human chorionic antigen. For the patient that was 69 years old, we only did CA-125 and carcinoma embryonic antigen (CEA). All patients had transabdominal ultrasound scans. The preoperative ultrasound scans documented huge unilocular cysts. Some cysts had fine septations, but there were no solid components or ascites. This indicated most likely a benign nature of the cysts. We do not do routine computerized tomography (CT) scans or magnetic resonance imaging (MRI) if the ultrasound scan findings are highly suggestive of a benign cyst, that is, unilocular cyst with no solid areas or thick septations and no ascites. However, some patients were referred with a CT scan. The mean size of the cysts as measured by preoperative ultrasound scans was 25.8 cm (range 20–42 cm). The tumor markers were all normal.

The mean operative time was 104 minutes (range 76–134 minutes). The mean volume of fluid drained from the cysts was 5200 mL (range 1000–12000 mL). Four patients had serous cystadenoma and one had mucinous cystadenoma.

Three trocars were used in all patients except one in whom four trocars were used as she had laparoscopic-assisted vaginal hysterectomy (LAVH). All patients tolerated the procedure well. There were no intraoperative or postoperative complications.

The blood loss was minimal, and all patients were discharged on the next postoperative day. The postoperative outpatient follow-up visit was arranged within 3-4 weeks from the surgery, and all the patients had no wound complications and have returned to their usual daily activities.

## 4. Discussion

Laparoscopic surgery has represented a major improvement in surgery recently because of its better magnification, reduced invasiveness, and shorter hospitalization.

 It is considered the gold standard treatment for small to moderate size ovarian cysts, but when confronted with extremely large and apparently benign cysts, only few surgeons advocate laparoscopic management due to technical difficulties like space constraints. Also, there is fear of cyst rupture and spillage of malignant cells.

Nowadays there is increasing evidence that huge ovarian cysts can be managed by laparoscopy. 

Large benign ovarian cysts are usually of serous or mucinous variety and almost always require resection due to their big size and associated symptoms [12].

Most adnexal masses are benign with malignancy found in only 7%–13% of premenopausal women and 8%–45% of postmenopausal patients [13]. The incidence of unsuspected ovarian cancer at laparoscopy has been shown to be only 0.04% [14].

Proper patients selection is mandatory to minimize the risk of draining malignant masses. Previous reports indicate that meticulous clinical and ultrasound examinations of ovarian cysts can exclude most cases of ovarian malignancies [15]. The addition of tumor markers levels and intraoperative cyst inspection prior to the drainage of the cyst should reduce this risk further.

Previous reports described preoperative ultrasound-guided drainage of the cysts; we prefer to drain the cyst intraoperatively after inspection of the cyst external surfaces during laparoscopy [10].

There is still no consensus for the size limitation of ovarian cysts decided to be a contraindication for laparoscopic management. The only thing needed is expertise in laparoscopic surgery and proper selection of patients. With advancing techniques and availability of experts in gynecological endoscopy, it is possible to remove giant cyst laparoscopically. Unfortunately, there is no randomized trials available regarding the management of giant ovarian cyst for more than twenty centimeter; only few case series and case reports are available.

We report here five cases of huge ovarian cysts managed successfully by laparoscopy. We hope that this case series will add to increase the evidence of laparoscopic techniques in the management of huge ovarian cysts.

## Figures and Tables

**Table 1:** Patients' characteristics and operative details.

Patients	1	2	3	4	5	Mean
Age (years)	69	19	20	23	22	
Maximum diameter (cm)	20	20	42	22	25	25.8
Operation time (minutes)	106	76	134	100	105	104
Fluids drained (mL)	1000	1500	12000	6000	5500	5200
Number of ports	4	3	3	3	3	
Pathology	Serous cystadenoma	Serous cysadenoma	Mucinous cysadenoma	Serous cysadenoma	Serous cysadenoma	
Procedure performed	LAVH + Bilateral salpingo-oopherectomy	Ovarian cystectomy	Salpingo-oopherectomy	Salpingo-oopherectomy	Salpingo-oopherectomy	

LAVH: laparoscopic-assisted vaginal hysterectomy.
